# Graded effects of unregulated smooth muscle myosin on intestinal architecture, intestinal motility and vascular function in zebrafish

**DOI:** 10.1242/dmm.023309

**Published:** 2016-05-01

**Authors:** Joshua Abrams, Zev Einhorn, Christoph Seiler, Alan B. Zong, H. Lee Sweeney, Michael Pack

**Affiliations:** 1 Department of Medicine, Perelman School of Medicine, University of Pennsylvania, Philadelphia, PA 19104, USA; 2 Department of Physiology, Perelman School of Medicine, University of Pennsylvania, Philadelphia, PA 19104, USA

**Keywords:** Myosin, Zebrafish, Intestine, Smooth muscle

## Abstract

Smooth muscle contraction is controlled by the regulated activity of the myosin heavy chain ATPase (Myh11). Myh11 mutations have diverse effects in the cardiovascular, digestive and genitourinary systems in humans and animal models. We previously reported a recessive missense mutation, *meltdown* (*mlt*), which converts a highly conserved tryptophan to arginine (W512R) in the rigid relay loop of zebrafish Myh11. The *mlt* mutation disrupts myosin regulation and non-autonomously induces invasive expansion of the intestinal epithelium. Here, we report two newly identified missense mutations in the switch-1 (S237Y) and coil-coiled (L1287M) domains of Myh11 that fail to complement *mlt*. Cell invasion was not detected in either homozygous mutant but could be induced by oxidative stress and activation of oncogenic signaling pathways. The smooth muscle defect imparted by the *mlt* and S237Y mutations also delayed intestinal transit, and altered vascular function, as measured by blood flow in the dorsal aorta. The cell-invasion phenotype induced by the three *myh11* mutants correlated with the degree of myosin deregulation. These findings suggest that the vertebrate intestinal epithelium is tuned to the physical state of the surrounding stroma, which, in turn, governs its response to physiologic and pathologic stimuli. Genetic variants that alter the regulation of smooth muscle myosin might be risk factors for diseases affecting the intestine, vasculature, and other tissues that contain smooth muscle or contractile cells that express smooth muscle proteins, particularly in the setting of redox stress.

## Abstract (precis)

**Editors' choice:** Two newly identified *Myh11* gene missense mutations discovered in a zebrafish enhancer-suppressor mutagenesis screen are reported. The mutations disrupt myosin regulation and ATPase activity in a graded fashion, and this correlated with their effects on intestinal and vascular physiology.

## INTRODUCTION

Smooth muscle in the vertebrate intestine is arranged in discrete layers surrounding the epithelium (Sanders et al., 2012). An outer layer, the tunica muscularis, comprises longitudinal and circular sublayers that shorten and narrow the intestinal lumen during peristaltic contractions. An inner layer present in mammals, the muscularis mucosa, controls fine movements of the epithelium. Predictably, mutations that disrupt smooth muscle contractile function have pronounced effects on digestive physiology and are a cause of heritable digestive disease (Gauthier et al., 2014; He et al., 2008; Thorson et al., 2014; Wang et al., 2010; Wangler et al., 2014).

Beyond their role in contraction, smooth muscle cells contribute to tissue homeostasis. Airway and vascular smooth muscle secretes cytokines and growth factors that modulate neighboring endothelial and epithelial cells (Lee et al., 2006; Seidel et al., 2009; Wang et al., 2011; Yang et al., 2014). Intestinal smooth muscle cells secrete many of these signaling molecules; however, their effects have not been demonstrated *in vivo* (Lin et al., 2014; Salinthone et al., 2004; Shi and Sarna, 2005; Wehner et al., 2010). Myofibroblasts are another contractile cell type residing within the intestinal stroma (Mifflin et al., 2011) that communicate with epithelial cells via biochemical signaling pathways (Andoh et al., 2007; Powell et al., 2011; Shaker et al., 2014; Zacharias et al., 2011). A subset of myofibroblasts lies in close proximity to the epithelium and it has long been suspected that they, and nearby smooth muscle, modulate epithelial function via physical signaling mechanisms. Supporting this idea, the adhesion, proliferation and migration of cultured intestinal epithelial cells is altered by changes in substrate rigidity, compressive force or mechanical strain (Chaturvedi et al., 2008, 2011; Craig et al., 2008; Gayer and Basson, 2009; Kovalenko et al., 2012; Krndija et al., 2010; Whitehead et al., 2008).

In previous work, we showed that physical signals arising from smooth muscle cells influence the behavior of adjacent epithelial cells in developing zebrafish larvae. Larvae homozygous for a missense mutation in the smooth muscle myosin heavy chain [*meltdown* (*myh11^W512R^*; hereafter referred to as *mlt*)] undergo invasive remodeling of the epithelium (Seiler et al., 2012; Wallace et al., 2005a). The single-amino-acid substitution in the Myh11 rigid relay loop disrupts regulation of myosin ATPase activity. This induces slow tonic contraction of newly formed intestinal smooth muscle and, in turn, non-cell-autonomously activates a feed-forward redox signaling pathway that induces the formation of matrix-degrading protrusions within the basal plasma membrane of adjacent epithelial cells. The protrusions, known as invadopodia, generate basement membrane gaps through which the invasive epithelial cells migrate into the tissue stroma. Because the invasive cells do not detach from neighboring epithelial cells, the mutant larvae develop large cysts that cause lethal obstruction of the posterior intestine by 10-12 days post-fertilization (dpf).

Heterozygous *mlt* mutants develop normally and have a normal lifespan. Unexpectedly, we previously found that the suppressive effects of the wild-type *myh11* allele can be overridden by drugs that generate oxidative stress (Seiler et al., 2012). The response of the heterozygotes to drug-induced stress argues that stress arising from endogenous sources, such as can occur during tissue injury or disease, might induce a similar response in humans who are heterozygous for germline or somatic *MYH11* mutations that disrupt myosin regulation. Supporting this idea, two independent studies identified a high frequency of somatic *MYH11* mutations in individuals with a hereditary form of colorectal cancer (Alhopuro et al., 2008; Vickaryous et al., 2008).

Here, we present additional evidence supporting the idea that mutations that alter smooth muscle myosin regulation can have clinically relevant effects on tissue physiology. We report the identification and characterization of two *myh11* missense mutations that fail to complement the original *mlt* mutation. Biochemical analyses confirmed that both mutations disrupt myosin regulation. Larvae that were homozygous for either of the non-complementing mutations developed normally; however, oxidative stress induced cell invasion in both, and the invasive response correlated with the degree to which myosin regulation was altered. A comparable effect on intestinal motility and vascular function was detected in *mlt* and one of the newly identified mutants that was studied. Collectively, these findings argue that *MYH11* coding variants could be risk factors for intestinal and vascular disorders in humans, and that their pathogenesis might be linked to periodic redox stress.

## RESULTS

### Forward genetic screen identifies previously unknown mutations that induce cell invasion in *mlt* heterozygotes

**Fig. 1.**
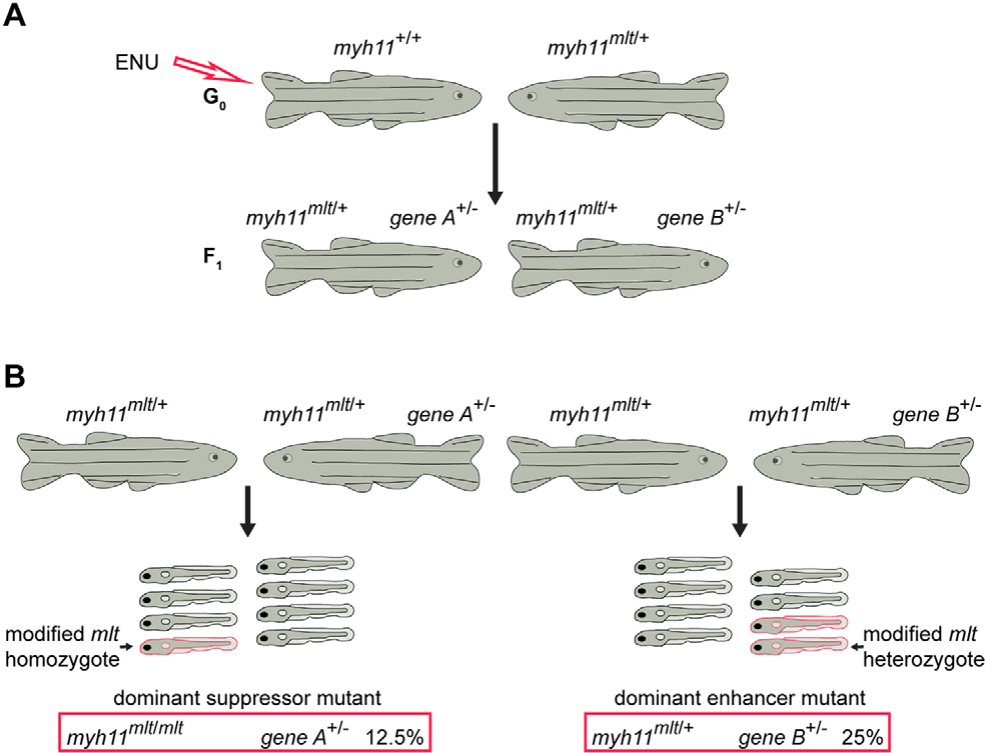
**Schematic overview of the *mlt* dominant modifier screen.** (A) Mutagenized wild-type adult fish were mated to *mlt* heterozygotes (*myh11^mlt/+^*). F1 *mlt* heterozygotes are heterozygous for a large number of ENU-induced mutations, here indicated by genes ‘A’ and ‘B’. (B) Random matings of F1 *mlt* heterozygotes carrying rare modifier mutations. Fully penetrant dominant suppressor mutations reduce the frequency of larvae with the typical *mlt* phenotype from 25% to 12.5% of total larvae. Fully penetrant dominant enhancer mutations induce a *mlt* phenotype in 25% of total larvae. Red larvae represent *mlt* homozygotes and modified heterozygotes.

We performed a genetic modifier screen to identify additional components of the epithelial redox signaling pathway activated by unregulated myosin in *mlt* mutants (Wallace et al., 2005a; Seiler et al., 2012). Adult male (F0) fish were treated with the mutagen ethylnitrosourea (ENU) (Dosch et al., 2004) and then mated with *mlt* heterozygotes (Fig. 1A). F1 heterozygous *mlt* fish (*myh11^mlt/+^*) were raised to sexual maturity and inbred to identify mutations that either enhanced or suppressed intestinal cell invasion in F2 *mlt* progeny (Fig. 1B). Progeny from 500 F1 pairs derived from two F0 parental fish were examined (∼1000 mutagenized genomes). Two independent F1 matings generated F2 larvae with a partial *mlt* phenotype in addition to the expected percentage (25%) of sibling larvae with the typical *mlt* phenotype. This phenotypic distribution suggested the presence of a single enhancer mutation in each family rather than a suppressor mutation.

**Fig. 2.**
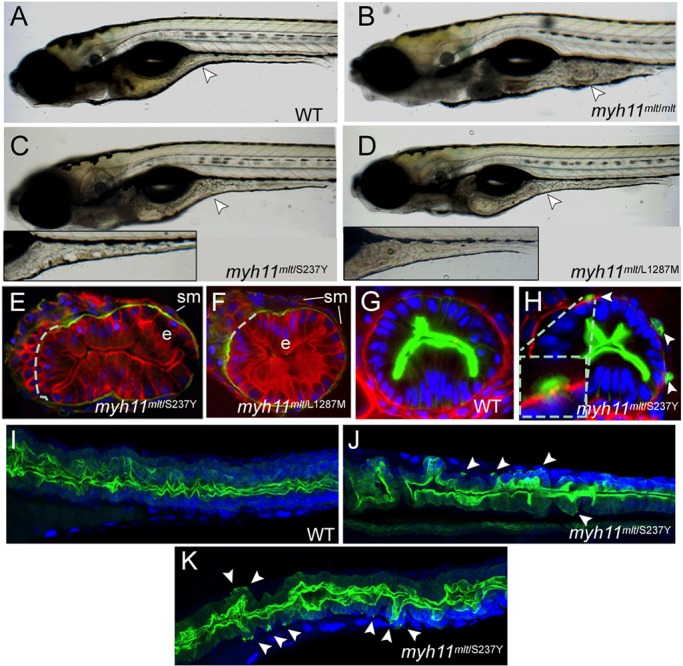
**Modifier mutations induce cell invasion in *mlt* heterozygotes.** (A-D) Lateral views of live 6-dpf larvae. Arrowhead points to the mid-intestine. (A) A wild-type (WT) larva. (B) A homozygous *mlt* larva in which there is invasive expansion of the mid- and posterior intestine. (C) A *mlt/*S237Y compound heterozygote. Arrowhead points to a region of altered intestinal morphology. Inset shows a more severely affected *mlt/*S237Y compound heterozygote. (D) *mlt/*L1287M compound heterozygote. Mild and moderate (inset) phenotypes are shown as in C. (E,F) Histological section through the posterior intestine of 6-dpf *mlt/*S237Y and *mlt/*L1287M compound heterozygotes following their immunostaining with anti-Keratin (red) and anti-Laminin-1 antibodies (green). Dashed line indicates predicted position of basement membrane disrupted by the invasive epithelial cells; e, intestinal epithelium; sm, smooth muscle. (G,H) Histological section through the posterior intestine of 3-dpf *Tg(miR194-Lifeact-GFP)* wild-type and sibling *mlt/*S237Y larvae following immunostaining with anti-GFP (green) and anti-Laminin-1 (red) antibodies, showing GFP-labeled invadopodia (arrowheads) of epithelial cells extending through gaps in the basement membrane. Inset shows a higher-magnification view. (I-K) Confocal projections through the mid- and posterior intestine of 4-dpf *Tg(miR194-Lifeact-GFP)* wild-type and sibling *mlt/*S237Y (*myh11^mlt/S237Y^*) larvae following anti-GFP immunostaining (green). Arrowheads point to invadopodia protruding from the basal epithelial cell plasma membrane in the *mlt/*S237Y mutants. Blue, DAPI-stained nuclei.

Molecular genotyping confirmed this prediction: all larvae with the modifier phenotype were heterozygous, rather than homozygous, for the original *mlt* allele. The two newly identified modifier mutations (*myh11^S237Y^* and *myh11^L1287M^*; discussed below) induced similar intestinal phenotypes in *mlt* heterozygotes. In both compound mutant larvae (*myh11^mlt/S237Y^* and *myh11^mlt/L1287M^*), the contour of the mid and posterior intestine appeared irregular compared with the wild-type intestine; however, in both, the severity of the phenotype varied (Fig. 2A-D). For the first modifier mutation (*myh11^S237Y^*), 37% of 70 F2 heterozygotes mated with *mlt* generated mild and moderately affected compound mutant larvae (∼1:2 ratio), whereas the remainder generated moderately affected mutants. For the second modifier mutation (*myh11^L1287M^*), 33% of 27 F2 heterozygotes generated mild and moderately affected compound mutant larvae (∼1:2 ratio), with the remainder generating moderately affected mutants. The percentage of larvae with the partial *mlt* phenotype derived from these matings was 17.3% and 18.9% (*n*=7964 and 2341 total larvae examined from matings of *myh11^S237Y/+^* and *myh11^L1287M/+^* F2 fish mated with *mlt*, respectively). These phenotypic distributions are consistent with incomplete penetrance of recessive modifier mutations.

Histological analyses of compound mutant larvae revealed the hallmarks of epithelial invasion in both compound heterozygotes, thus confirming that the mutations functioned as *mlt* enhancers (Fig. 2E,F). In wild-type larvae, the intestine is composed of a single-layered epithelium that is always separated from the underlying circular and longitudinal smooth muscle layers by a thin basement membrane but very little connective tissue (Wallace et al., 2005a,b). Invasive epithelial cells in *mlt* and the compound mutant larvae breach the laminin-rich basement membrane and extend into the underlying tissue stromal layer.

Immunostaining of one of the compound heterozygotes (*myh11^mlt^^/S237Y^*) that expressed the Lifeact-GFP transgene in the intestinal epithelium showed invadopodia-like actin-rich protrusions at sites of gaps in the basement membrane several hours before cell invasion was evident in live larvae, as in *mlt* homozygotes (Seiler et al., 2012), whereas these protrusions were never detected in the wild-type intestine (Fig. 2G-K). These findings suggested that the modifier mutations, when combined with *mlt*, altered myosin regulation and activated the redox signaling loop that drives invadopodia formation and cell invasion in *mlt* homozygotes (Seiler et al., 2012).

### The modifier mutations encode newly identified *myh11* variants

Genetic-mapping experiments were used to determine the chromosomal location of each mutation. F1 fish that were heterozygous for one of the modifier mutations and the original *mlt* allele were mated with the polymorphic WIK wild-type strain so that the frequency of meiotic recombination could be scored in the F2 ‘mapping’ fish. Unexpectedly, none of the clutches from these F2 incrosses contained F3 larvae with the original *mlt* phenotype and the modifier phenotype. This strongly suggested that the modifier loci were genetically linked to the original *myh11^mlt^* mutation, and this was subsequently confirmed in matings between F2 fish from the S237Y (*n*=432) and L1287M (*n*=265) mapping lines with unrelated *mlt* heterozygotes. These experiments placed the S237Y and L1287M modifier mutant loci within 0.46 and 0.75 cM of the *mlt* mutation.

**Fig. 3.**
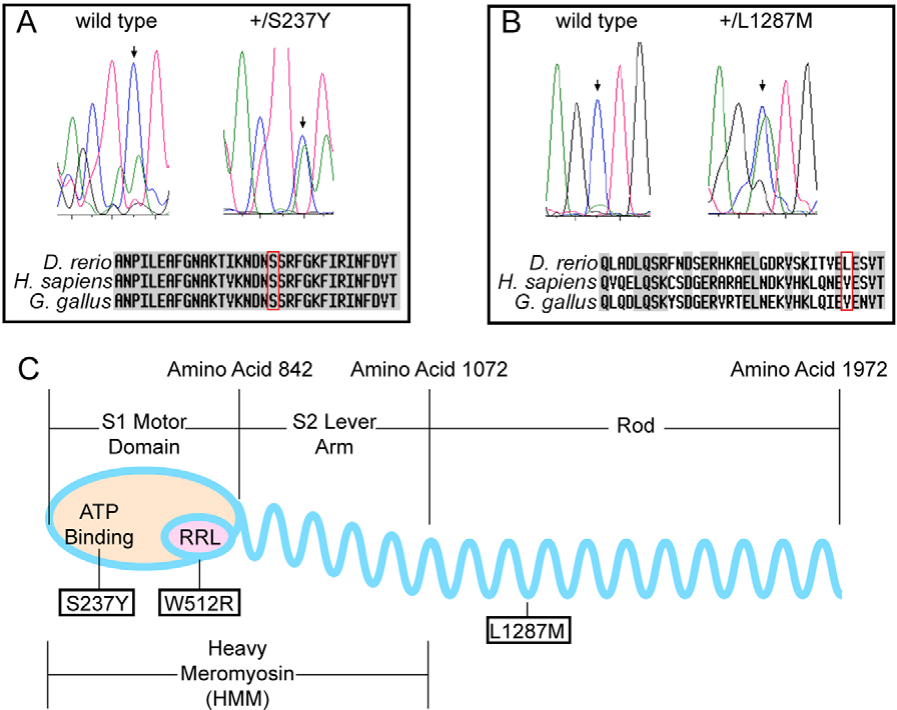
***mlt* modifier mutations encode newly identified *myh11* alleles.** (A,B) Sequencing of intestinal cDNA from modified *mlt* heterozygous larvae identifies distinct *myh11* mutations. Arrows indicate the location of the cytosine-to-adenine transversion mutations that change serine 237 to tyrosine (S237Y) and leucine 1287 to methionine (L1287M). Both amino acid substitutions are outlined in red boxes. Comparable amino acid sequences of human and chicken Myh11 proteins are indicated. (C) Cartoon depicting conserved domains within the MYH11 protein and the corresponding locations of the zebrafish *myh11* mutations.

To determine whether either of the modifier mutations resided within the *myh11* coding region, we sequenced the 5922-bp *myh11* cDNA sequence derived from the intestinal mRNA of compound heterozygous larvae. Of the four potential Myh11 isoforms (arising from two splice variants), we obtained intestinal cDNAs encoding the SM1A and SM1B isoforms, as previously reported (Wallace et al., 2005a). The sequence data from the cDNAs showed that, in addition to the original expected *mlt* mutation, a single newly identified *myh11* missense mutation was present in each compound heterozygote (Fig. 3A,B). One mutation converted a highly conserved serine to tyrosine (S237Y) within the switch-1 region of the myosin head (Fig. 3C). Switch-1 is one of three domains that form the nucleotide (ATP)-binding pocket, and it plays a role in transmitting information between the nucleotide- and actin-binding regions of myosin (Decarreau et al., 2011; Kintses et al., 2007). The second mutation, L1287M, was located within the coiled-coil dimerization domain of the myosin tail. The corresponding amino acid residue in human MYH11 is valine, which, like leucine in zebrafish Myh11, is hydrophobic and has a short aliphatic side chain. Using the COILS program (www.ch.embnet.orgsoftwareCOILSform.html), the methionine substitution was predicted to enlarge a small region of discontinuity in the coiled-coil domain of the myosin rod (Fig. S1).

### The S237Y and L1287M mutations disrupt myosin regulation

The *mlt* mutation (W512R) is located within the Myh11 rigid relay loop, a domain that establishes a communication pathway between three different regions of the myosin head: the ATP- and actin-binding regions and the converter/lever arm (Yengo et al., 2000). During smooth muscle contraction, conformational changes of the lever arm move the myosin head on the actin filament, which generates contractile force (Sweeney and Houdusse, 2010). Myh11 containing the *mlt* W512R amino acid substitution has constitutive, low-level ATPase activity that is independent of myosin phosphorylation or the presence of actin, presumably because the arginine substitution interferes with lever arm movement (Wallace et al., 2005a). Constitutive ATPase activity of the mutant Myh11 is predicted to disrupt cross-bridge cycling in *mlt* smooth muscle, effectively generating a state of low-level tonic contraction, which we predicted would alter the physical state of the intestinal stromal compartment. Time-lapse imaging and biochemical experiments in *mlt* homozygotes are consistent with this prediction (Seiler et al., 2012).

**Fig. 4.**
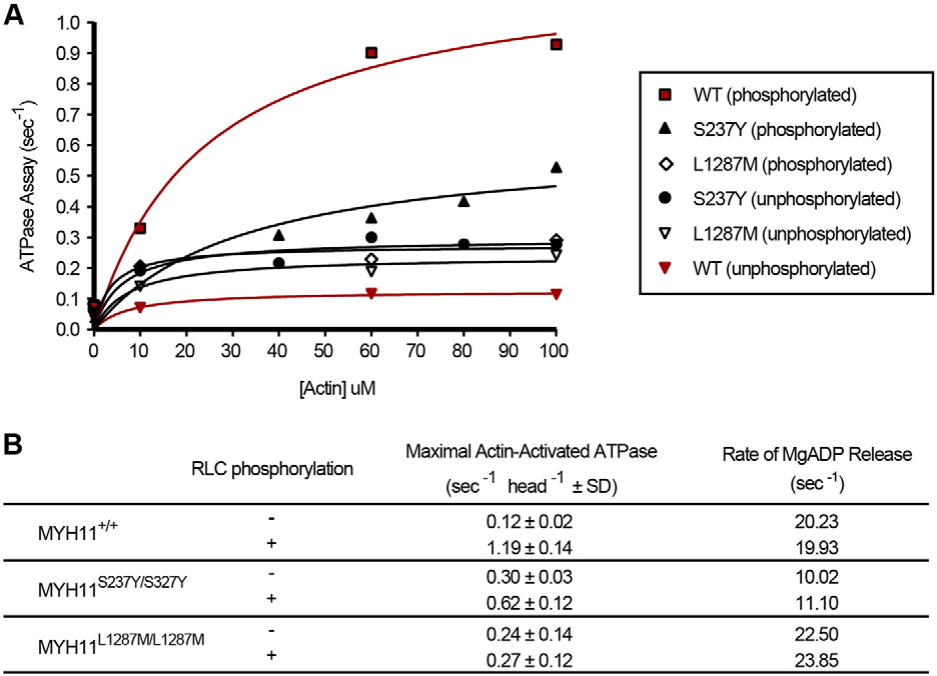
***mlt* modifier mutations alter myosin regulation.** (A) Steady-state (normalized) ATPase activity of wild-type (WT) HMM, S237Y HMM and full-length L1287M. Data from phosphorylated and unphosphorylated assays are shown. There is low level activity of the S237Y and L1287M myosins in the absence of phosphorylation. Phosphorylation increases the activity of wild-type greater than it does S237Y myosins, but does not alter the activity of L1287M myosin. (B) Table showing values for maximum ATPase and ADP release for phosphorylated and unphosphorylated WT, S237Y and L1287M myosins. RLC, regulatory light chain.

Biochemical analyses of human myosins engineered to contain the orthologous S237Y or L1287M mutations showed that each amino acid substitution disrupted myosin regulation, similar to *mlt* (Fig. 4A,B). The ATPase activity of non-phosphorylated S237Y in the heavy meromyosin fragment (HMM), and of full-length MYH11 that contained the L1287M mutation in the myosin tail, was increased compared with the corresponding wild-type MYH11, and was similar to the ATPase activity of non-phosphorylated *mlt-*HMM (Wallace et al., 2005a). However, unlike *mlt*, both required the presence of actin. Phosphorylation doubled the maximum ATPase activity of S237Y-HMM, although it was still less than wild-type-HMM, whereas phosphorylation had only a small effect on the ATPase activity of L1287M, similar to *mlt*. Although we have not determined how the mutations disrupt myosin regulation, they likely interfere with interactions within the folded regulatory complex, which requires interactions between both the heads and the rods of the myosin dimer (Rovner et al., 2006; Walcott and Warshaw, 2010; Wendt et al., 2001).

In addition to ATPase activity, we also measured the rates of ADP release from the mutant myosins (Fig. 4B). ADP release was reduced in S237Y-HMM in the absence of phosphorylation, but slightly increased when the mutant protein was phosphorylated. It was essentially unchanged in L1287M-HMM. An effect of S237Y on ADP release is not surprising, given its position in the switch-1 region of the nucleotide-binding pocket. Slowed ADP release prolongs strong binding to actin, which will reduce the speed of muscle shortening.

### S237Y and L1287M *myh11* mutations do not disrupt intestinal architecture

Larvae derived from matings of S237Y homozygotes and matings of L1287 compound heterozygotes all had normal intestinal morphology and survived beyond 14 dpf. Approximately 80% of S237Y homozygotes survived to adult stages (*n*>50 larvae reared from homozygous S237Y matings), whereas none of the L1287M homozygotes survived to adult stages (*n*≥100 expected mutants from heterozygous matings). The cause of early mortality of L1287M mutants, which occurred during the transition from larval to juvenile stage, was not determined.

Immunostainings using antibodies directed against epithelial and basement membrane markers confirmed the normal intestinal morphology: invasive cells were not detected in histological sections of the intestine in either mutant (data not shown). We also looked for the presence of invadopodia, because we previously detected them in the absence of cell invasion in wild-type larvae that express a constitutively active *Src* transgene in the intestinal epithelium (Seiler et al., 2012). Invadopodia were also not detected in homozygous S237Y that express the Lifeact-GFP transgene in intestinal epithelial cells (data not shown).

### S237Y and L1287M *myh11* mutants have graded responses to redox stress

**Fig. 5.**
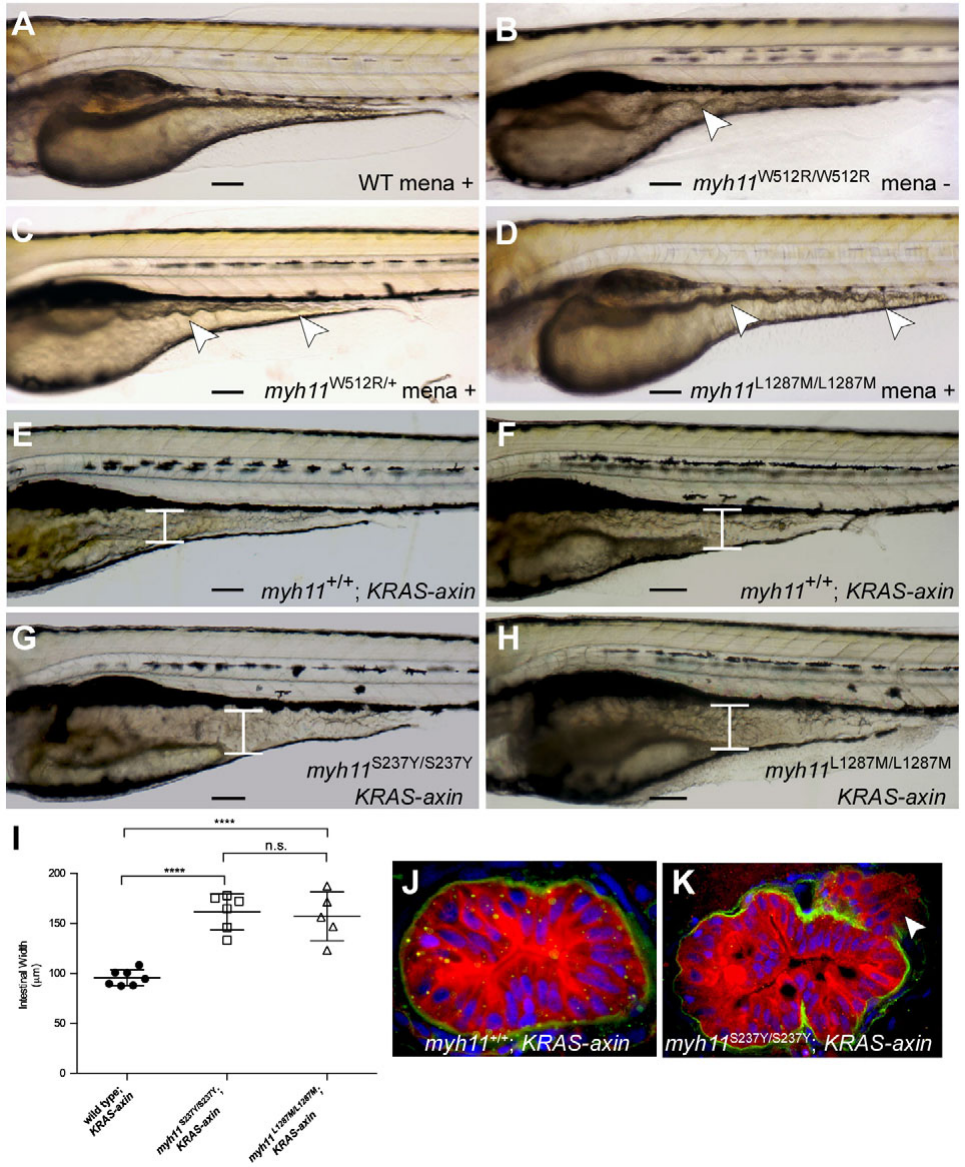
***mlt* modifier mutants are sensitized to oxidative stress.** (A-D) Lateral views of 82-hpf wild-type (WT), homozygous *mlt*, heterozygous *mlt* and L1287 homozygous larvae. Wild-type (A), *mlt* heterozygote (C) and L1287M homozygote (D) larvae treated with menadione (1.5 μM; mena). Morphological changes of the mid- and posterior intestine that are characteristic of epithelial invasion (arrowheads) are seen in the *mlt* larva (B) and the menadione-treated *mlt* heterozygote and L1287M homozygote (C,D). In all three mutant larvae, the ventral intestine appears to extend into the yolk. Intestinal morphology is normal in the wild-type larva (A). (E-H) Lateral views of 6-dpf KRAS-axin larvae that either have homozygous wild-type *myh11* (E,F), S237Y (G) or L1287M (H) alleles. The intestine is expanded in KRAS-axin larvae as a result of epithelial hypertrophy (brackets, E,F). Further expansion as a result of epithelial invasion is seen in menadione-treated KRAS-axin S237Y and L1287M homozygotes (brackets, G,H), as previously reported for menadione-treated KRAS-axin *mlt* heterozygotes (Seiler et al., 2012). (I) Quantification of intestinal width in KRAS-axin compound-mutant larvae treated with menadione compared with untreated siblings. ****P<0.001; n.s., not significant. Unpaired Student's *t*-test performed; mean±s.e.m. (J,K) Histologic analyses of immunostained larvae showing epithelial cell invasion in the intestine of a menadione-treated homozygous S237Y KRAS-axin larva (K) vs a wild-type KRAS-axin larva (J). Green, anti-Laminin-1; red, anti-Keratin; blue, DAPI-stained nuclei. Arrowhead (K) points to invasive cells. Scale bars: 100 µm.

In previous work, we identified a role for redox signaling in the invasive response of epithelial cells to unregulated myosin. We showed that redox-sensitive genes were upregulated in the intestine of *mlt* homozygotes, and that cell invasion could be induced in *mlt* heterozygotes by the drug menadione, which generates intracellular reactive oxygen species (ROS) when metabolized to an unstable semiquinone (Iyanagi, 1990; Seiler et al., 2012). We tested the response of compound heterozygous or homozygous S237Y and L1287M mutant larvae to menadione and found that intestinal morphology was unchanged in menadione-treated S237Y homozygotes, S237Y heterozygotes and L1287M heterozygotes (data not shown). The menadione treatment did, however, induce cell invasion in the L1287M homozygotes (21% of 237 larvae from matings of L1287M heterozygotes), as evidenced by intestinal morphology typically seen in *mlt* homozygotes, and *mlt* heterozygotes treated with menadione (Fig. 5A-D) (Seiler et al., 2012).

The invasive response of 3-dpf heterozygous *mlt* and homozygous L1287M mutants treated with menadione is lost at later developmental stages (5 dpf). Responsiveness of older *mlt* heterozygotes (5 dpf) is restored by co-activation of oncogenic signaling pathways that are frequently activated in colorectal cancers (KRAS, Wnt) (Seiler et al., 2012). This is demonstrated in 5-dpf menadione-treated *mlt* heterozygotes that express an activated form of human KRAS in the intestinal epithelium and are homozygous for an *axin1* loss-of-function mutation that activates the canonical Wnt signaling pathway [*Tg(miR194:eGFP-KRAS^G12V^); axin^tm213/tm213^;* *myh11^mlt^^/+^*; hereafter referred to as ‘KRAS-axin’ fish]. On their own, the mutant *KRAS* transgene and the *axin* mutation cause intestinal epithelial hyperplasia and increase epithelial cell proliferation, respectively, in zebrafish larvae, but do not cause cell invasion or neoplasia (Cheesman et al., 2011; Langenau et al., 2007; Seiler et al., 2012). KRAS-axin larvae that are homozygous for the wild-type *myh11* allele are not responsive to menadione.

To determine whether the S237Y mutation might sensitize larvae to oncogenic signaling, we treated heterozygous and homozygous S237Y KRAS-axin larvae with menadione at 5 dpf. 38% of menadione-treated larvae derived from the heterozygous S237Y KRAS-axin matings developed intestinal expansion typical of cell invasion previously observed in menadione-treated KRAS-axin *mlt* heterozygotes (Seiler et al., 2012) (Fig. 5E-G,I). 25% of these larvae were predicted to be S237Y homozygotes; thus, some S237Y heterozygotes might also be responsive to menadione. Cell invasion in the menadione larvae was confirmed with immunostainings of epithelial and basement membrane markers (Fig. 5J,K). The histological appearance most likely reflects the combined effect of cell invasion and increased cell proliferation driven by the *axin* mutation, along with constitutive smooth muscle contraction, which shortens the length of the intestine (Cheesman et al., 2011; Seiler et al., 2012). Identical morphological changes were detected in larvae derived from heterozygous L1287M KRAS-axin matings (Fig. 5H,I). This was expected, given the invasive response of 3-dpf homozygous L1287M larvae to menadione (Fig. 5D).

We next tested the response of mutant larvae to inhibition of the smooth muscle regulatory protein Caldesmon, which triggers cell invasion in *mlt* heterozygotes (Abrams et al., 2012; Seiler et al., 2012). In the non-phosphorylated form, the high-molecular-weight smooth muscle Caldesmon isoform inhibits contractile force. Phosphorylation relieves this inhibition. Caldesmon is prematurely phosphorylated in homozygous *mlt* larvae and in heterozygous *mlt* mutants with cell invasion induced by menadione (Seiler et al., 2012). The association between Caldesmon phosphorylation and cell invasion suggests that Caldesmon plays a causative role in the acquisition of the *mlt* phenotype. Consistent with this idea, Caldesmon knockdown, which is equivalent to phosphorylation when levels of non-phosphorylated protein are high (as in 3-dpf larvae), induces invasion in *mlt* heterozygotes, just like the menadione treatment (Seiler et al., 2012). In contrast to the *mlt* heterozygotes, Caldesmon knockdown using a previously validated isoform-specific morpholino (Abrams et al., 2012; Seiler et al., 2012) did not induce cell invasion in larvae that were either heterozygous or homozygous for either the S237Y or L1287M mutation (data not shown). This result argues that the *mlt* mutation uniquely alters interactions between actin and myosin in the smooth muscle cell contractile apparatus.

### Intestinal transit is reduced in S237Y and *mlt* homozygotes

**Fig. 6.**
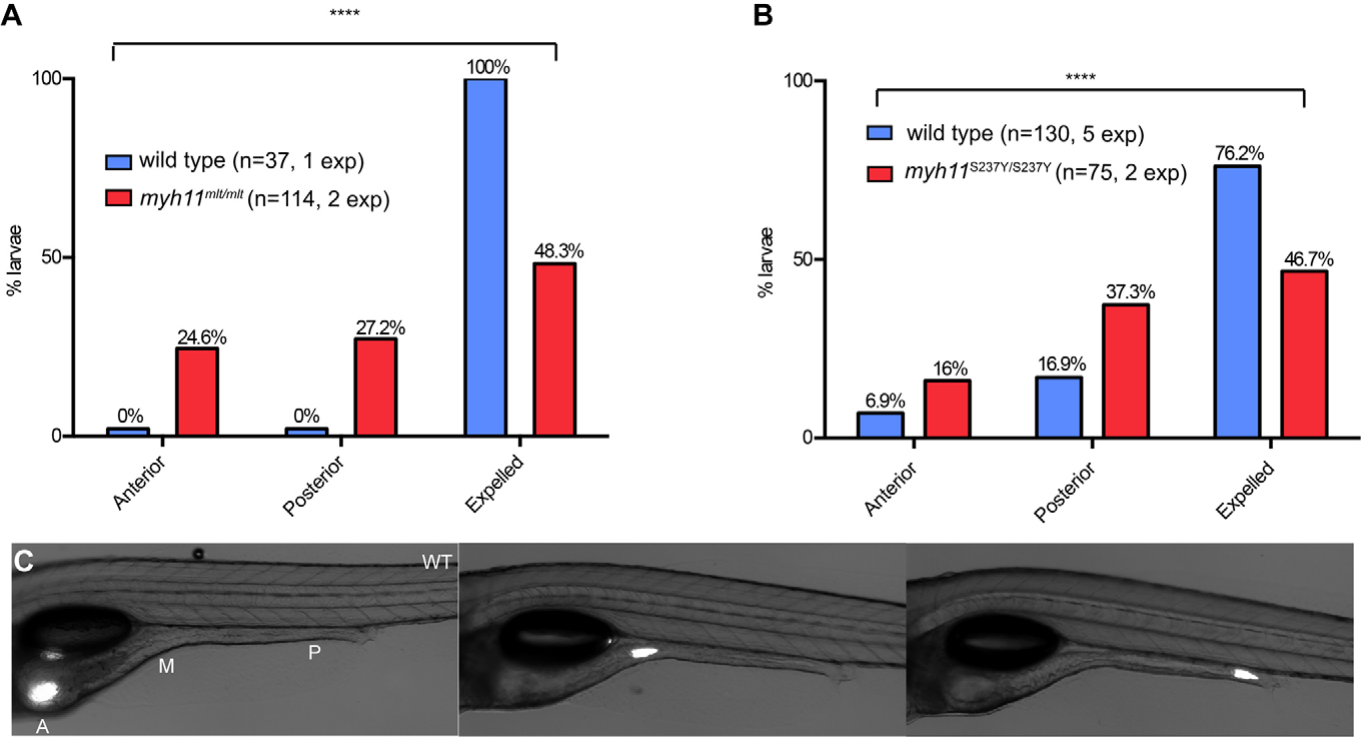
**Zebrafish *myh11* mutations alter intestinal transit.** (A,B) Intestinal transit as measured by the expulsion of fluorescent microspheres in wild-type and sibling homozygous *mlt* larvae injected with a *myh11* morpholino (MO) (A), and wild-type and sibling S237Y homozygous larvae (B). Anterior: beads remain in anterior intestinal bulb; posterior: beads remain in mid- and posterior intestine; expelled: no beads remaining in the intestine. ****P<0.001. Chi-squared test performed with 2 degrees of freedom. (C) Time lapse of a wild-type (WT) larva beginning when the fluorescent beads are present in the anterior intestine and followed until bead expulsion. A, M, P, location of anterior intestine, mid-intestine and posterior intestine, respectively.

Mutations that alter the function of *Mlck*, the kinase that phosphorylates the myosin regulatory light chain (RLC), disrupt intestinal motility in humans and in animal models (He et al., 2008; Wang et al., 2010). A recessive loss-of-function mutation in *MYH11* has also recently been cited as a cause of the heritable megacystis-microcolon-intestinal hypomotility syndrome (MMIHS) (Gauthier et al., 2014). To test whether the zebrafish *myh11* missense mutations reduced intestinal transit, we assayed the rate of expulsion of fluorescent beads ingested by the mutant larvae and their wild-type siblings (Abrams et al., 2012; Davuluri et al., 2010). We first studied *mlt* homozygotes rescued by transient antisense knockdown of the mutant Myh11 protein. In previous work, we showed that rescued *mlt* larvae, which are indistinguishable from wild-type siblings, have near normal levels of the mutant Myh11 protein beginning at 5 dpf (Wallace et al., 2005a). Studying rescued larvae therefore allowed us to assay motility in mutants with normal intestinal morphology, whereas, in non-rescued mutants, intestinal transit is blocked by large obstructing posterior cysts. Embryos derived from matings of adult *mlt* heterozygotes were injected with a previously validated *myh11* morpholino and were raised to 6 dpf. Larvae with normal intestinal morphology, which included wild-type, heterozygous mutants and rescued homozygous mutants, were fed fluorescent beads and bead transit was assessed the following morning via fluorescence microscopy (Fig. 6C). In these experiments (Fig. 6A), 24.6% of larvae from the *mlt* matings retained beads in the anterior intestine (*n*=28 of 114), 27.2% of larvae retained beads in the mid and posterior intestine (*n*=31 of 114), and the remainder (48.3%; *n*=55 of 114) expelled the beads 24 h after ingestion. Larvae derived from the wild-type mating (*n*=37 larvae) expelled all beads during this time period (Fig. 6A). Together, these findings suggest that all of the rescued homozygous mutant larvae and some heterozygous larvae had delayed transit. Supporting this, ten genotyped larvae that retained beads in the anterior intestine, a finding indicative of the most delayed transit, were all rescued homozygous *mlt* mutants. These findings are consistent with previous biochemical analyses predicting that the *mlt* mutant myosin would not support coordinated peristaltic contractions (Wallace et al., 2005a).

We next measured intestinal transit in S237Y homozygotes rather than L1287M homozygotes because a large percentage of the S237Y mutants survive as adults and are fertile. Larvae derived from matings of adult S237Y homozygous fish were compared with larvae derived from siblings that were homozygous for the wild-type *myh11* allele (Fig. 6B). 53.3% of S237Y homozygotes (*n*=40 of 75) expelled beads during an 8-h interval, compared with 76.2% of wild-type larvae (*n*=99 of 130). At 24 h, all S237Y homozygous larvae had expelled the beads (not shown). Thus, the S237Y mutation delayed transit, but its effect was less pronounced than with *mlt*.

### Vascular function is altered in S237Y and *mlt* homozygotes

Tonic contraction of vascular smooth muscle generates the isometric force needed to maintain and regulate blood pressure and blood flow. This function is in part related to the ability of vascular smooth muscle to generate contractile force with intermediate levels of RLC phosphorylation, and hence lower rates of ATP hydrolysis (Rovner et al., 2006; Walcott and Warshaw, 2010). This is commonly referred to as the latch state. Because sustained force generation in vascular smooth muscle is less dependent on myosin phosphorylation than in intestinal smooth muscle, we were interested in knowing whether the *myh11* mutations that disrupt myosin regulation altered vascular function. In addition, dominant mutations in *MYH11* are associated with a heritable human vascular disease, thoracic aortic aneurysm and dissection (TAAD), with or without patent ductus arteriosus (PDA) (Bee et al., 2012; Pannu et al., 2007; Renard et al., 2013; Zhu et al., 2006). *MYH11* mutations and copy-number variants have also been detected in individuals with sporadic TAAD (Milewicz et al., 2008; Prakash et al., 2010). It is not known whether any of the human *MYH11* mutations associated with TAAD alter myosin regulation.

**Fig. 7.**
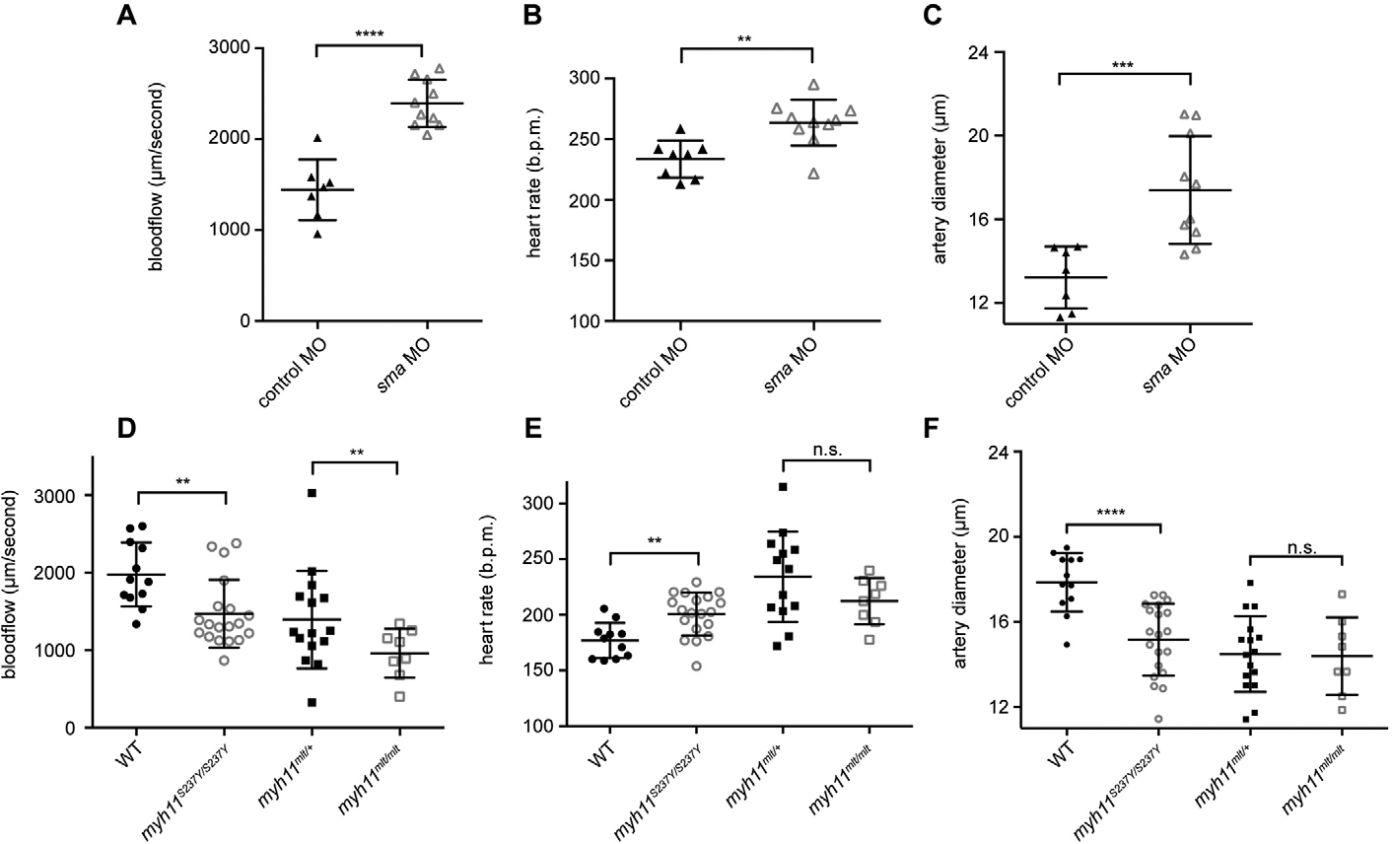
**Zebrafish *myh11* mutations alter vascular function.** (A-F) Recordings of blood flow (dorsal aorta), heart rate and aortic diameter in live 4-dpf larvae injected with control and smooth muscle actin (SMA; *acta2*) morpholinos (MO) (A-C). (D-F) Identical recordings from uninfected 6-dpf wild-type larvae (WT) and sibling S237Y homozygous mutants, and heterozygous *mlt* larvae along with *myh11*-MO-injected homozygous *mlt* larvae. ****P<0.0001; ***P<0.001; **P<0.01; n.s., not significant. SMA MO (A-C) and mutant (D-F) experiments performed independently. Unpaired Student's *t*-test performed. Mean±s.e.m.

To examine the effect of the zebrafish *myh11* mutations on vascular function, we calculated the rate of blood flow in the dorsal aorta of rescued *mlt* homozygotes (which have normal vascular anatomy in the intestine), S237Y homozygotes and their sibling wild-type larvae. As a Myh11-independent control, we first analyzed larvae injected with a previously validated antisense morpholino targeting smooth muscle actin (SMA; *acta2*), which blocks smooth muscle contraction through 4 dpf (Abrams et al., 2012; Seiler et al., 2012; Davuluri et al., 2010). Both the control and *acta2* morpholino injections caused mild developmental delay compared with uninjected control larvae, as seen in previous experiments (Seiler et al., 2012). Nonetheless, 4-dpf larvae deficient in *acta2* had increased blood flow in the aorta (Fig. 7A) and an increased diameter of the dorsal aorta (Fig. 7B), compared to larvae injected with a control morpholino. Heart rate was slightly increased in the SMA-deficient larvae, most likely in response to vasodilation, and this might have made a small contribution to increased blood flow (Fig. 7C). The effects of the SMA knockdown on vascular function, which are similar to the effects of treatment with the vasodilator sodium nitroprusside (Fritsche et al., 2000), support a role for smooth muscle in regulating vascular resistance in larval zebrafish.

We next examined the vascular parameters in *mlt* and S237Y mutants. For *mlt*, we studied larvae rescued by transient *myh11* knockdown to avoid potentially confounding effects caused by altered intestinal anatomy in the non-rescued homozygous mutants. 6-dpf larvae were analyzed to ensure sufficient recovery of Myh11 protein following morpholino knockdown. In contrast to the *acta2*-deficient larvae, in which aortic blood flow was increased as a result of decreased smooth muscle contraction, blood flow was reduced in both rescued *mlt* homozygotes and S237Y homozygous mutants (Fig. 7D, Movies 1-4). The effects on heart rate were variable and thus unlikely to have affected blood flow (Fig. 7E). Aortic diameter was reduced in S237Y homozygotes, but not in rescued *mlt* homozygotes, possibly because the wild-type pool included heterozygous mutants (Fig. 7F). Collectively, these data argue that both mutations increase vessel resistance, most likely as a result of reduced compliance caused by non-regulated smooth muscle contraction.

### *MYH11* variants detected via exome sequencing

**Fig. 8.**
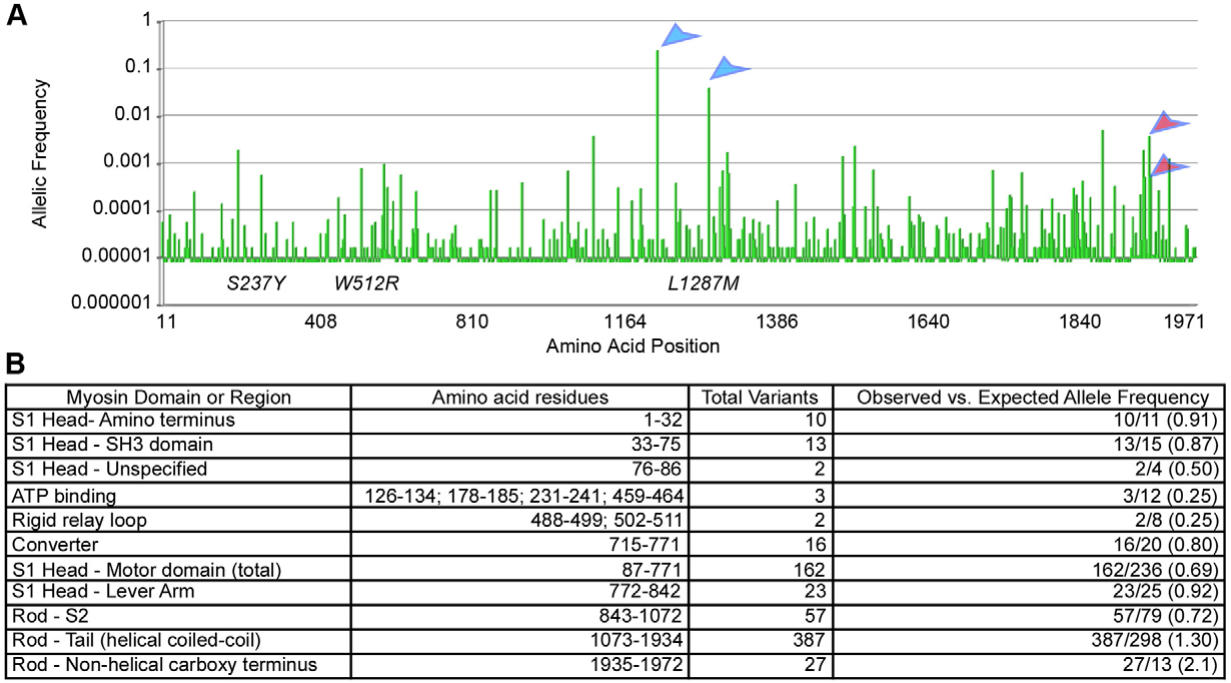
***MYH11* variants detected via exome sequencing.** (A) Bar graph depicting location and frequency of 682 *MYH11* variants retrieved from the ExAc server; the location of the *mlt* (W512R), S237Y and L1287M mutations is indicated. Blue arrowheads point to the location of the Ala1241Thr and Val1296Ala variants, which are likely to be benign substitutions, and pink arrowheads point to the C7 and C9 variants in the SM2 allele. (B) Table listing the predicted and observed number of variants in different domains and regions of human MYH11. Ratio of observed to predicted listed in parentheses.

To determine whether human orthologs of the zebrafish *myh11* mutations have been detected in genome-sequencing projects, we reviewed the location and frequency of *MYH11* variants listed in the Exome Aggregation Consortium (ExAc) browser (www.exac.broadinstitutie.org: July, 2015). We did not find homologs of the zebrafish *myh11* mutations represented in this population. The 682 variants listed were distributed across all the conserved myosin heavy chain domains, with the majority having a very low allele frequency (<10^−5^) (Fig. 8). Fewer variants were detected in the motor domain than predicted to occur by chance, which suggests that variants in this region of the protein might be deleterious (Fig. 8B). In contrast, a larger percentage of variants were present in the myosin tail and carboxy terminus. The allele frequencies of two variants in the tail domain (Ala1241Thr and Val1296Ala; blue arrowheads Fig. 8A) were high (25% and 4% of alleles, respectively) and thus are likely to be benign substitutions. Only eight other variants had an allele frequency greater than 0.1%. Interestingly, one of these was a deletion of a single cytosine residue within a mononucleotide repeat of eight cytosines (C8) present in the last exon of the *MYH11* SM2 isoform. This variant cDNA encodes an insertion of 90 amino acids in the carboxy terminus of the SM2 isoform (allele frequency 0.38%). The identical somatic mutation (C7) was detected in a percentage of patients with hereditary colorectal cancer by two independent groups and was shown to alter myosin regulation (Alhopuro et al., 2008; Vickaryous et al., 2008). A similar effect on myosin regulation was induced by another cancer-associated mutation that inserts 19 amino acids as a result of the insertion of a single cytosine in this mononucleotide repeat (C9). The allele frequency of this variant was 0.05%. The combined allele frequency of the two variants affecting the C8 mononucleotide repeat (C7 and C9 alleles; red arrowheads Fig. 8A), which both alter myosin regulation, was 0.44%. This implies that, in the populations represented on the ExAc server, ∼1 in 114 persons will be heterozygous for a *MYH11* allele that disrupts myosin regulation. Whether any of the other missense or loss-of-function variants will disrupt regulation cannot be predicted, but it is likely that some will, based on the analyses of other mutations in other Myosin II proteins.

## DISCUSSION

In previous work, we showed that smooth muscle myosin carrying a missense mutation in the Myh11 rigid relay loop, *myh11^mlt^*, had non-regulated, actin-independent ATPase activity. Unexpectedly, zebrafish larvae that were homozygous for this mutation, *mlt*, developed invasive expansion of the intestinal epithelium that caused lethal intestinal obstruction (Wallace et al., 2005a). Here, we show that a milder form of this phenotype can be induced in larvae carrying *myh11* mutations that have distinct effects on myosin regulation, but that on their own do not affect intestinal architecture. These findings support the idea that the cell invasion phenotype displayed in the *myh11* mutants is triggered by changes in the resting mechanical properties of the intestine, and provide additional evidence that genetic variants that alter myosin regulation can have physiologically and clinically important consequences in humans.

The three *myh11* mutations discussed in this study, *mlt* (W512R), L1287M and S237Y, induce a graded invasive response within the intestinal epithelium. The most robust response occurs with *mlt*. Epithelial invasion occurs spontaneously in 3-dpf homozygous *mlt* mutants, and can be induced in heterozygotes by oxidative stress (menadione treatment) or by enhancing actomyosin interactions (Caldesmon knockdown) (Seiler et al., 2012). In older heterozygotes (5 dpf), co-activation of oncogenic signaling is required to trigger stress-induced invasion. The epithelium of L1287M and S237Y mutants is less responsive than in *mlt*. Homozygotes develop normally through larval stages. However, the invasive phenotype can be induced by oxidative stress: L1287M larvae are more sensitive and invasion occurs at both early (74 hpf) and later (5 dpf; with oncogenic signaling activated) time points, whereas, in S237Y mutants, invasion is triggered only at the later time point, when oncogenic signaling is activated.

Biochemical studies reported here show that the responsiveness of the epithelium to the *myh11* mutations correlates with their effect on myosin regulation. *In vitro*, non-phosphorylated W512R-HMM (*mlt*) continuously hydrolyzes ATP in the absence of actin (Wallace et al., 2005a). This is predicted to cause the myosin head to remain adherent to actin in a strong binding state for prolonged periods of time, thus altering both resting tension within the intestinal wall and the response of smooth muscle to contractile stimuli (supported by previous *in vivo* imaging studies) (Seiler et al., 2012). Non-phosphorylated S237Y and L1287M Myh11 proteins have unregulated ATPase activity, similar to *mlt*; however, each requires actin for activation. Thus, although the S237Y and L1287M myosins are expected to undergo unregulated, continuous cross-bridge cycling, they are predicted to place less of an energetic burden on the cells than the *mlt* myosin. As a result, the resting physical characteristics of the smooth muscle in these mutants are predicted to be less pronounced than in *mlt*. The S237Y mutant is also expected to reduce force generation in response to contractile stimuli, given its effect on ADP release; however, it will likely generate more force than the L1287M myosin (but less than wild type), because the ATPase activity of S237Y-HMM increases with RLC phosphorylation. This could account, at least in part, for why adult S237Y homozygotes survive, whereas L1287M homozygotes die as juveniles, and why S237Y homozygous larvae are less responsive to oxidative stress and have only modestly reduced intestinal transit.

*MYH11* coding variants and *MYH11* copy-number variants cause heritable and sporadic vascular disease, including familial TAAD (with or without patent ductus arteriosus), sporadic TAAD, and other sporadic human vascular disorders (Bee et al., 2012; Kuang et al., 2011; Pannu et al., 2007; Zhu et al., 2006). Current models predict that these mutations are either haploinsufficient or function as dominant-negatives to disrupt smooth muscle contraction and or mechanosensing. Over time, this weakens the wall of the ascending aorta, which is under substantial mechanical stress throughout an individual's life (Humphrey et al., 2014). It is not known whether any of the *MYH11* TAAD alleles affect myosin regulation, although a single rare variant associated with sporadic TAAD does not (Kuang et al., 2012; Bellini et al., 2015). Thus, it cannot easily be predicted whether vascular defects caused by other TAAD *MYH11* alleles involve activation of the redox signaling loop that causes cell invasion in zebrafish.

The findings reported here provide a potential link between vascular diseases, such as TAAD, and myosin regulation in smooth muscle: we show that *mlt* and S237Y mutations reduced aortic blood flow in the 6-dpf larvae. This argues that the human orthologs of these and related *myh11* mutations reduce vascular compliance, which is an early finding in individuals who are heterozygous for dominant familial TAAD mutations. To date, we have not detected overt aortic pathology in any of the zebrafish *myh11* mutants, but this might reflect differences in cardiovascular anatomy and physiology in fish and humans. In addition, we have not excluded the possibility that aortic disease contributes to lethality in L1287M homozygotes. Supporting this possibility, *MYH11* mutations in two TAAD families are close to L1287M: one encodes a 24-amino-acid in-frame deletion between amino acids 1241 and 1274 (R1241_L1264del) (Zhu et al., 2006); the other encodes two adjacent missense mutations (L1264P and R1275L) (Pannu et al., 2007). Going forward, it will be interesting to determine whether germline *MYH11* variants that are strongly linked to cardiovascular disease affect myosin regulation.

*MYH11* variants have also been detected in individuals with hereditary non-polyposis colorectal cancer (HNPCC), those with a form of microsatellite instability (MSI) colorectal cancer, and in cases of sporadic cancer with stable microsatellites (MSS cancer) (Alhopuro et al., 2008; Vickaryous et al., 2008). The most common of these somatic mutations either add or delete one nucleotide from eight consecutive cytosines (C8) in the last exon of the *MYH11* SM2 isoform, which is expressed in all smooth muscle and plays an important regulatory role in the intestine and bladder (Chi et al., 2008). Myosin containing the C7 and C9 MYH11 variants, which both elongate the protein, showed non-regulated contraction in *in vitro* assays, and, like *mlt* and L1287M, were not responsive to phosphorylation. Thus, these acquired mutations could play a role in cancer progression, a prediction that is consistent with their presence in invasive cancers but not in non-malignant polyps (Vickaryous et al., 2008). Indeed, when one considers the high allele frequency of the C7 and C9 variants (0.44%) along with the frequency of other missense and loss-of-function *MYH11* variants reported on the ExAc server (4.36%; excluding two common variants), a large number of individuals might be germline carriers of *MYH11* alleles that alter myosin regulation.

In conclusion, the work reported in this study, combined with our previous work on the *mlt* mutation, argues that *MYH11* variants that alter myosin regulation are risk factors for stress-induced changes in tissues homeostasis. In the digestive tract, this could promote intestinal cancers to become invasive, which is required for metastasis. In the setting of inflammation or ischemia, non-regulated myosin might impede or prevent proper regenerative responses of the epithelium, thus altering digestion, gut immunity or even the microbiome. The *MYH11* variants could also predispose individuals to stress-induced changes in contractile function that could contribute to the development of poorly understood motility disorders that are exacerbated by inflammatory states, such as irritable bowel syndrome and gastroparesis (Beatty et al., 2014; Parkman, 2015). Supporting an inducible model of tissue injury, vascular damage in mice engineered to carry the R247C mutation, a common variant associated with non-sporadic TAAD that alters myosin contractility, but not its regulation, causes more neointima formation than in wild-type mice (Kuang et al., 2012). The R247C mutant mice are also susceptible to hypertension-induced changes in vascular wall structure that are also seen in human TAAD (Bellini et al., 2015). Additional testing in mouse models will help determine the role of other common *MYH11* variants in sporadic cardiovascular disease.

Constitutive myosin activation will change the mechanical properties of the intestine, which normally must sense and respond to normal changes in wall tension caused by peristaltic contraction. The findings presented here argue that genetic variants targeting components of the mechanosensing rheostats that regulate these homeostatic pathways might also be expected to alter epithelial behavior. A similar mechanism has recently been proposed to explain the pathogenesis of TAAD, which, in addition to mutations that target contractile proteins (including *MYH11*), is caused by mutations in structural proteins and force transducers in the aortic wall (Humphrey et al., 2014).

## MATERIALS AND METHODS

### Zebrafish husbandry, mutagenesis and morpholino injections

Maintenance and breeding of adult zebrafish (Tübingen, AB and WIK strains) were performed as previously described (Wallace et al., 2005a). All experiments were performed in accordance with guidelines of the University of Pennsylvania Institutional Animal Care and Use Committee. Zebrafish embryos and larvae were raised at 28°C in E3 medium and were staged by age and morphological criteria (size of yolk extension and pigment pattern around yolk extension). The transgenic lines used have been previously described [*Tg(miR194-mCherry)*; *Tg(miR194-Lifeact-*GFP*)*; *Tg(sm22alpha-GFP)*] (Seiler et al., 2012). Expression of mutant human *KRAS* fused to GFP (*GFP-KRAS^G12V^*) in the intestinal epithelium was driven by the *miR194* promoter described by Seiler et al. (2012). The *GFP-KRAS^G12V^* transgene was a generous gift from Steven Leach (Memorial Sloan Kettering Cancer Institute, New York, NY). Zebrafish *axin1^tm13^* mutants were obtained from the Zebrafish International Resource Center (Eugene, OR). ENU mutagenesis was performed on males from Tübingen and AB strains according to the scheme outlined by Dosch et al. (2004). Surviving males (F_0_) were mated with *meltdown* (*mlt*) heterozygotes. F1 progeny heterozygous for the *mlt* mutation were raised to sexual maturity and intercrossed to generate F2 larvae and then were screened at 3 dpf and 6 dpf for enhancer or suppressor phenotypes of the previously characterized *mlt* intestinal phenotype (Seiler et al., 2012; Wallace et al., 2005a). For morpholino injections, larvae were injected with 2-10 ng of previously validated morpholinos targeting *acta2*, the high-molecular-weight isoform of *caldesmon*, and *myh11* (Abrams et al., 2012; Davuluri et al., 2010; Seiler et al., 2012; Wallace et al., 2005a).

### Intestinal transit assay and video recordings of intestinal smooth muscle contraction

Wild-type and mutant larvae were fed paramecia in E3 media containing fluorescent latex beads (Fluoresbrite™ YG2.0; Polysciences, Warrington, PA; 5 µl beads per 2 ml of E3 media) for 2-6 h. Larvae were then screened for fluorescence in the anterior intestine, washed and transferred into media containing only paramecia, and then monitored for bead expulsion using a fluorescent dissecting microscope (Olympus, MVX-10). To generate time-lapse movies of intestinal contraction, larvae were anesthetized with equal amounts of tricaine in E3 media (64 mg l^−1^) and mounted in 3% methylcellulose for imaging. Images were collected for 5-15 s intervals for 2-6 min using an RGB Vision digital camera (Roper Scientific Photometrics, Tucson, AZ) and Image-Pro Plus Version 6.0 software (Media Cybernetics, Bethesda, MD).

### Vascular recordings

Larvae (4 dpf and 6 dpf) were anesthetized with equal amounts of tricaine in E3 media (64 mg l^−1^) and mounted in 3% methyl cellulose. Blood flow in the dorsal aorta was recorded for 5 s using a high-speed camera (Motionpro 2000; Redlake, Tucson, AZ) at 250 frames per second (fps) with a 640×480 resolution. Images of the heart were recorded for 5 s at 125 fps with a 512×512 resolution, and the heart rate was determined by monitoring arterial contractions. Quantification of the arterial blood flow rate was performed by tracking individual intra-luminal red blood cells over a distance of 500 μm using ImageJ software. To determine the flow rate, the following calculation was used: *V_μ__m/s_=*(*fps*)×(*pr*)×(*d*)/(*f*), where *V*=flow rate (μm/s), *fps*=movie frame rate, *pr*=ratio of pixels to microns (i.e. microns per pixel), *d*=distance tracked (in pixels) and *f*=number of frames counted. The diameter of the dorsal aorta was measured using the ImageJ polygon tool to trace a blood vessel segment the length of six somites; this area measurement was then converted to width (in μm) using the calculation of width=area/major axis. Heart rate was measured by tracking five cardiac cycles and recording the total number of frames that elapsed; this was then converted to beats per minute (bpm) using the equation: *R_heart_=*(*n*)×(*fps*)/*f*, where *R*=heart rate (bpm), *n*=number of cardiac cycles tracked, *fps*=movie frame rate and *f*=number of frames elapsed.

### Immunostaining and histological analyses

Larvae were anesthetized with 0.1 mg/ml tricaine, fixed in 4% PFA/PBS, washed in PBST (PBS+0.1% Tween), dehydrated in methanol and stored at −20°C. For whole-mount staining with anti-laminin and anti-cytokeratin antibodies, larvae were washed in PBST and permeabilized by a 15-min Proteinase K digestion (100 µg/ml in PBST). They were then rinsed in PBST and post-fixed in 4% PFA/PBST. The skin above the trunk and intestine was removed using fine forceps. Larvae were stained with antibody in 10% goat serum/PBST. The laminin antibody (Sigma #L-9393) was used at 1:50 or 1:200 dilution; the cytokeratin antibody (Thermo Scientific clone AE1/AE3, MS-343-PO) was used at 1:100 dilution. For Lifeact-GFP labeling, larvae were stained with Alexa-Fluor-488-conjugated anti-GFP (Molecular Probes/Invitrogen A-11073) at 1:1000 dilution. Secondary antibodies were labeled with Alexa-Fluor-568 or -488 (Molecular Probes/Invitrogen). Histological analyses were performed as described (Wallace et al., 2005a).

### Drug treatment

Larvae were bathed in 1.5 µM Menadione (MP Biomedicals) in E3 media for 3 h (3-dpf larvae) or 5 h (5-dpf larvae) and were scored immediately thereafter for the intestinal cell invasion phenotype as described (Seiler et al., 2012).

### Genotyping and cloning of zebrafish *myh11* cDNA

Intestines manually dissected from 5-dpf larvae were homogenized in Trizol (Sigma-Aldrich, St Louis, MO) and total RNA was collected following the manufacturer's protocol. First-strand cDNA was synthesized using the SuperScript™ III Reverse Transcriptase (Invitrogen, Carlsbad, CA) according to the manufacturer's procedure. When necessary for sequencing, amplified cDNA fragments were cloned into the pGEM^®^-T Easy Vector (Promega, Madison, WI). Sequencing was performed on cDNA obtained from the intestine of compound heterozygotes (*myh11^mlt/S237Y^* and *myh11^mlt/L1287M^*) and analyzed manually for individual single-nucleotide polymorphisms (SNPs) using MacVector 12.5.

### Construction of mutant human smooth muscle myosin heavy chain proteins for *in vitro* assay

The full-length cDNA for human *MYH11* (SM1A isoform) was truncated at the codon for threonine 1775 [thus generating the heavy meromyosin (HMM) fragment], after which a glycine plus FLAG peptide (DYKDDDDK) was appended to facilitate purification. Site-directed mutagenesis was performed using QuikChange XLII kit (Stratagene) to introduce the S237Y mutation into the same construct. The constructs were subcloned into the baculovirus transfer vector p2Bac (Invitrogen). Protein expression and purification were performed as previously described (Sweeney et al., 1998). The HMM construct had been previously subcloned into the baculovirus transfer vector pVL 1393 (Invitrogen). Baculovirus expression was used to produce HMM fragments of smooth muscle myosin after infection of an insect cell line (Sf9) with recombinant baculovirus (Sweeney et al., 1998). Mutagenesis of full-length *MYH11* containing the corresponding L1287M amino acid substitution (V1289M in human SMA1 MYH11 isoform) was performed by site-directed mutagenesis by Mutagenex (Hillsborough, NJ).

### Myosin ATPase assay and transient kinetic assays

The actin-activated ATPase activity assay was performed at 25°C in buffer 20/20 (KCl 20 mM, Mg^2+^ 5 mM, EGTA 1 mM, MOPS 20 mM pH 7.0), ATP 1 mM final concentration and actin concentration ranging from 0 to 150 μM. Actin was purified from rabbit skeletal muscle and stabilized by phalloidin. Phosphorylation of HMM wild-type and mutant constructs was performed as previously described (Yang and Sweeney, 1995). Both unphosphorylated and phosphorylated forms of wild-type and mutant human smooth muscle myosin HMM and full-length constructs were assayed at 0.2 nM final concentration. Curves were fitted with Kaleidagraph software™. Triplicate assays were performed with three different preparations of each protein. Transient kinetic measurements (ADP release from actomyosin) were made in buffer 20/20 at 250/20 at 25med with Photophysics SX.18MV stopped-flow instrument following previously published protocols (De La Cruz et al., 1999). Assays were performed with three different preparations of each protein.
